# The Utilization of Graphene Oxide in Traditional Construction Materials: Asphalt

**DOI:** 10.3390/ma10010048

**Published:** 2017-01-07

**Authors:** Wenbo Zeng, Shaopeng Wu, Ling Pang, Yihan Sun, Zongwu Chen

**Affiliations:** State Key Laboratory of Silicate Materials for Architectures, Wuhan University of Technology, Wuhan 430070, China; zwb0212@whut.edu.cn (W.Z.); wusp@whut.edu.cn (S.W.); sunyh@whut.edu.cn (Y.S.); chenzongwu@whut.edu.cn (Z.C.)

**Keywords:** graphene oxide, asphalts, penetration, softening point, rheological properties

## Abstract

In the advanced research fields of solar cell and energy storing materials, graphene and graphene oxide (GO) are two of the most promising materials due to their high specific surface area, and excellent electrical and physical properties. However, they was seldom studied in the traditional materials because of their high cost. Nowadays, graphene and GO are much cheaper than before with the development of production technologies, which provides the possibility of using these extraordinary materials in the traditional construction industry. In this paper, GO was selected as a nano-material to modify two different asphalts. Then a thin film oven test and a pressure aging vessel test were applied to simulate the aging of GO-modified asphalts. After thermal aging, basic physical properties (softening point and penetration) were tested for the samples which were introduced at different mass ratios of GO (1% and 3%) to asphalt. In addition, rheological properties were tested to investigate how GO could influence the asphalts by dynamic shearing rheometer tests. Finally, some interesting findings and potential utilization (warm mixing and flame retardants) of GO in asphalt pavement construction were explained.

## 1. Introduction

Stable graphene, as a two-dimensional single sheet, was firstly discovered by Andre Geim and Konstantin Novoselov [1]. Graphene plays a very important role in nano-materials due to its excellent physical and electrical properties [2,3], and in polymer fields, many applications of graphene have been reported in that it can significantly improve the properties of polymers [4]. However, graphene is not suitable to be produced on a large scale due to the limited existing producing technologies [5]. Graphene oxide (GO) is the precursor of graphene, which is more convenient to be produced at a large scale. It also has a two-dimensional structure and inherits the beneficial properties of graphene.

So many researchers have investigated the possibilities of modifying polymers with GO. Chen used GO in epoxy/silica composites and found that it could greatly enhance the mechanical properties of epoxy resin [6]. Yongjin found that the conductivity, thermal, mechanical, and rheological properties of poly(methylmethacrylate) were improved by introducing GO [7]. As reported in Zhenhai’s paper, GO could significantly increase the modulus and tensile strength of butadiene–styrene–vinyl pyridine rubber [8]. Moreover, various polymers, such as carboxylated acrylonitrile butadiene rubber [9,10], polypropylene [11], and poly(arylene disulfide) [12], that were modified by GO to enhance their physical properties. In addition to the increasing physical properties, a gas barrier feature is also endowed with GO-modified polymers. GO sheet is impermeable for different gases (He, O_2_, CO_2_, etc.) due to the high potential energy barrier, which provides many potential applications, such as inhibiting oxidation and aging of polymers [13].

Asphalt is considered as a kind of organic mixture, which is made by the distillation of crude oil. Just like polymers, asphalt also shows viscous-elastic properties. It has been widely applied in the pavement constructions for its comfortableness and smoothness. However, it is also facing many problems that may shorten the serving time of pavements. The poor elastic modulus and high viscous modulus in summer may lead to rutting on the road surface. On the contrary, a high elastic modulus and poor viscous modulus in winter may lead to surface cracking. Moreover, another of the most serious challenges of asphalt roads is oxidation aging [14,15,16]. Many researchers have investigated traditional anti-aging additives (graphite, montmorillonite) to improve the physical properties and ease the aging process that happens in the serving period [17,18,19]. Moreover, some novel materials (TIO_2_, CeO_2_, Layered Double Hydroxides) have been studied for their enhancing strength and anti-aging characters [20,21,22]. So far, modifiers of asphalt is one of the most popular research fields, and more novel materials should be investigated to produce the ideal asphalt.

GO, as described before, is a potential material to improve the physical properties and anti-aging ability of asphalt. Thus, in this paper, GO was introduced into asphalt to investigate how it influences the asphalt properties and whether GO could improve the anti-aging properties of asphalt. In this paper, the softening point and penetration were used to evaluate the physical properties. Rheological properties were also carried out to study the effects of GO on asphalt. In the meantime, the anti-aging property was also investigated by these three tests. Finally, the reason why GO does not show significant improvement on the anti-aging property was explained and some interesting applications (warm mixing and flame retardants) were discussed.

## 2. Experiments

### 2.1. Materials

GO was bought from Heng Qiu Graphene Technology (Suzhou) Co., Ltd. (Suzhou, China).

Two types of asphalts, with 60/80 pen grade and 80/90 pen grade, were applied in this investigation. These asphalts were supplied by KOCH Asphalt Co., Ltd. (Ezhou, China). The physical properties of two asphalts were listed in Table 1. 70A and 90A are abbreviations of asphalts with 60/80 pen grade and 80/90 pen grade, respectively.

### 2.2. Aging Procedure

70A, 90A, and GO-modified asphalts were firstly aged by a thin film oven test (TFOT) at 163 °C. Then the residues from the TFOT were aged by a pressure aging vessel (PAV) test at 95 °C and 2.1 MPa air pressure. TFOT was executed in accordance with ASTM D1754, and the PAV test in accordance with ASTM D6521.

### 2.3. Preparation Procedure of GO-Modified Asphalt

In general, there are three ways to prepare the GO modified polymer. The first one is the solution-mixing method. However, complete evaporation of the solution is the crucial problem with this method [23]. The second preparation method is in situ polymerization. However, in situ polymerization also faces the evaporation problem because the solution state also has to be applied when in situ polymerization is conducted [24]. The last method is the melting method. In the melting method, mechanical mixing is applied to disperse GO into molten polymers. It is a very convenient, economical, and environmentally friendly method to produce GO-modified polymers on a large scale [25]. In this paper, it is hard to evaporate carbon disulfide, trichloroethylene, or xylene if these solutions were used to prepare the GO-modified asphalt. Thus, the melt method was selected to produce the GO-modified asphalts.

Firstly, the molten asphalt was prepared as the matrix at 135 °C. Then 1% and 3% (mass percentage) of GO were introduced into the two asphalts, respectively. Moreover, in order to produce a good dispersed GO-modified asphalt, a high shear mixer was used to shear and mix the GO in the asphalt. Finally, the GO-modified asphalt was prepared.

**Figure 1:**
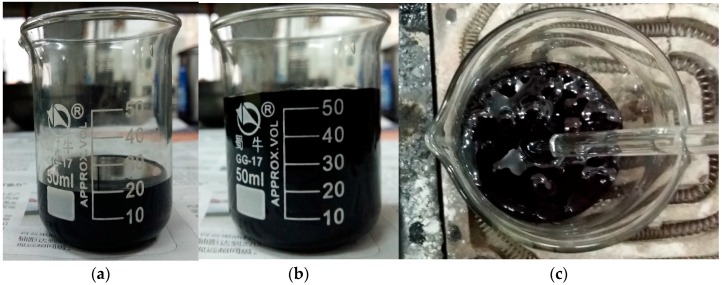
Boiling effect phenomenon. (**a**) Volume of 1% GO-modified asphalt before heating; (**b**) volum of 1% GO-modifed asphalt after heating; and (**c**) gas released.

In the preparation process, an interesting phenomenon was found. When heating the GO-modified asphalt, a large amount of gas was released above 115 °C. Figure 1 shows the gas was released in the GO-modified asphalt and the volume of modified asphalt was increased by almost three times. Then the expansive volume was reduced to its initial state after cooling. In this paper, the boiling reaction is defined for this phenomenon because it is similar to boiling water.

### 2.4. Test Method

#### 2.4.1. Dynamic Shear Rheometer (DSR) Tests

The DSR (MCR101, Anton Paar, Graz, Austria) was used to investigate the rheological properties of both GO-modified asphalts and base asphalts before and after TFOT and PAV aging. Both high and low temperature sweep tests were performed under strain-controlled mode with a constant frequency of 10 rad/s. The testing temperature ranged from −10 °C to 30 °C and 30 °C to 80 °C with temperature increment of 2 °C per minute. When the temperature is below 30 °C, plates with 8 mm diameter and a 2 mm gap were applied. When the temperature is above 30 °C, plates with 25 mm diameter and a 1 mm gap were used.

#### 2.4.2. Softening and Penetration Tests

The penetration (25 °C, 0.1 mm) and softening point (°C) of the binders were tested in accordance with standards ASTM D564 and ASTM D3626, respectively.

#### 2.4.3. Pyrolysis-Gas Chromatography/Mass Spectrometry (Py-GC/MS) Test

Pyrolysis-gas chromatography/mass spectrometry is a very useful method to investigate the volatile gases of organic materials at certain temperatures. In this research, a Py-GC/MS machine (Agilent 6890N/5975, Agilent, Santa Clara, CA, USA) was used to determine what kinds of gases were released from GO modified asphalt. In this test, GO modified asphalt was pyrolyzed at 115 °C. Then the released gases were analyzed by gas chromatography and mass spectrometry to investigate the gas components.

### 2.5. Experimental Plan

**Figure 2:**
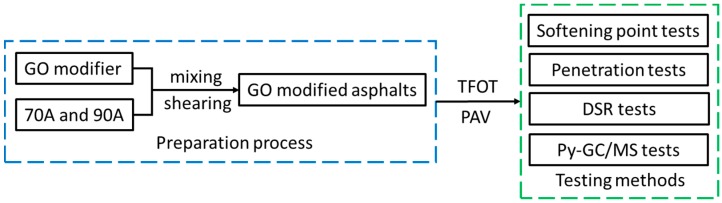
Experimental plan.

The experimental plan is shown in Figure 2. Firstly, the GO modifier and base asphalts were mixed and sheared to prepare GO-modified asphalts. Then, TFOT and TFOT+PAV aging were applied to simulate the aging process. Finally, softening point tests, penetration tests, DSR tests, and Py-GC/MS tests were used to evaluate how GO would influence the properties of asphalts.

## 3. Results and Discussion

### 3.1. Softening Point Tests

**Figure 3:**
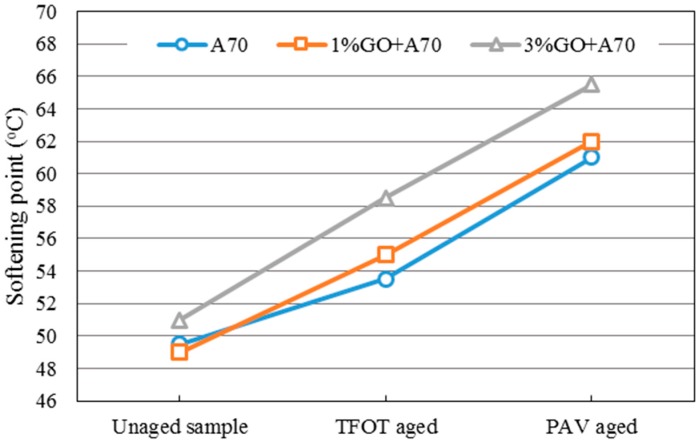
Softening point of GO-modified 70A and its matrix before and after aging.

**Figure 4:**
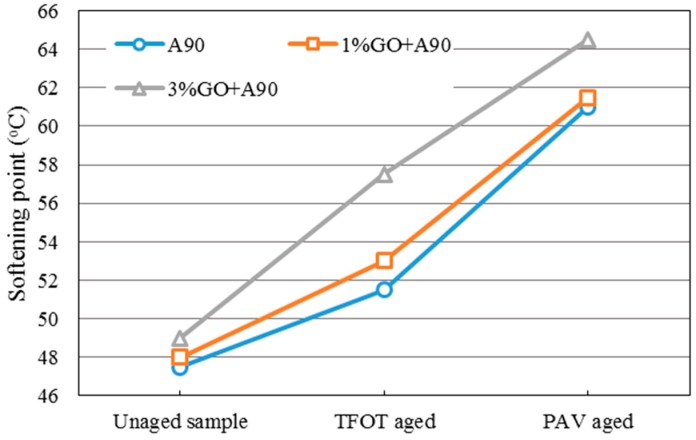
Softening point of GO-modified 90A and its matrix before and after aging.

The softening point could be used to evaluate the soft and hard degree of asphalt. Once asphalt is oxidized, the softening point of it increases. Thus, a greater increase in the softening point indicates a more serious aging index of the asphalt after aging. Figure 3 and Figure 4 showed the changing tendency of the softening point of GO-modified 70A, GO-modified 90A, and their matrix before and after aging.

From both graphs, it was obvious that the softening point (SP) of GO-modified asphalts and the base asphalts were almost the same before aging when the GO content is 1%. In addition, 1% GO-modified asphalts showed greater increments compared to both base asphalts after TFOT aging process. However, softening points of both modified asphalts with 1% GO were almost the same as the base asphalt after the PAV aging process. This phenomenon could be attributed to the boiling reaction when the temperature was above 115 °C. Gas was released in the TFOT aging process in which the aging temperature was 163 °C. The GO-modified asphalts were boiled by gas in the TFOT oven and it resulted in a similar stir action in the inner part of asphalts. So more air was exposed to asphalt, more serious aging index of asphalt happened. Moreover, the aging temperature was only 90 °C in the PAV aging process, at which temperature the boiling reaction would not occur. The sheet structure and impermeable property of GO could block the permeation of air. It could slow down the oxidation rate of asphalt and lead to a similar SP value of both modified and base asphalts after PAV aging.

When the content of GO was 3% in 70A and 90A, the softening point was much higher than that of matrix asphalt before and after aging, compared to 1% GO-modified asphalt. Before aging, it was easily understandable that inorganic powder (GO) enhanced the hardness of asphalt. In addition, with more GO content, the boiling reaction was more severe in the TFOT aging process. Thus, a much higher SP value of 3% GO-modified asphalt is shown in Figure 3 and Figure 4 after TFOT aging. For PAV aging, the increasing slope of the 3% modified asphalt between TFOT and PAV aging was slightly smaller than that of the 1% modified asphalt. This might mean the anti-aging property was improved slightly.

### 3.2. Penetration Tests

Penetration presents a degree of consistency for asphalt, indicating the ability of resisting shear failure. Under certain conditions, it could reflect the relative softness and hardness degree of asphalt. A larger penetration indicates that asphalt is softer; conversely, a smaller penetration means that asphalt is harder.

**Figure 5:**
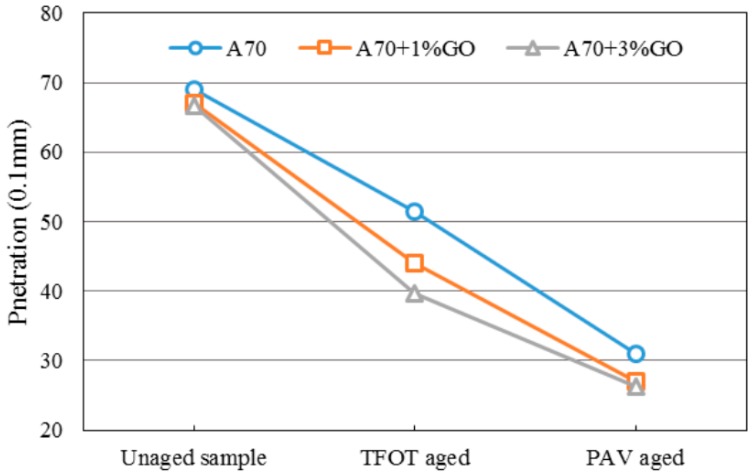
Penetration of GO-modified 70A and its matrix before and after aging.

**Figure 6:**
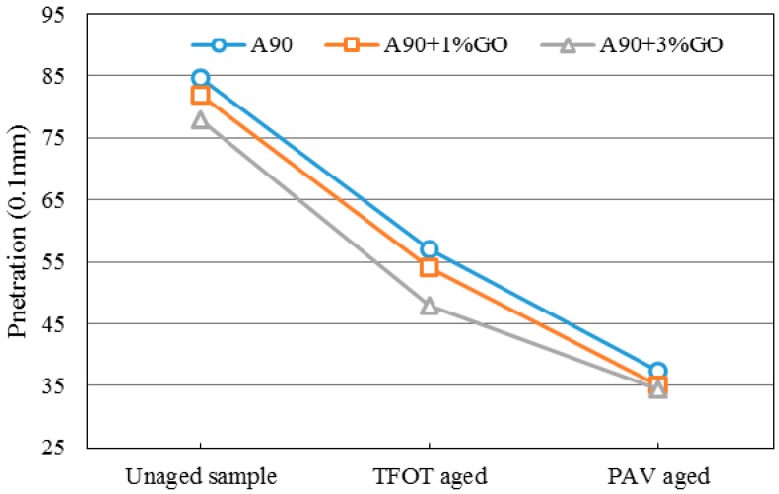
Penetration of GO-modified 70A and its matrix before and after aging.

Figure 5 and Figure 6 display the penetration changes of modified and base asphalts before and after aging. With the addition of GO, the penetration (Pen) of modified asphalts all decreased slightly before aging, and the Pen decrement of GO-modified asphalt was significantly larger and slightly smaller than base asphalt after TOFT aging and PAV aging, respectively. The changing tendency of Pen was the same as the SP.

The penetration and softening point results showed GO might promote the anti-aging property of asphalts. However, the anti-aging effects were not so obvious.

### 3.3. Temperature Sweep

#### 3.3.1. High-Temperature Sweep

In high temperature conditions, the viscosity of asphalt reduces and its flowability increases. This is the main cause of asphalt pavement rutting damage at high temperatures. Thus, the larger complex modulus means the better high temperature performance, which leads to a better ability to resist rutting in a high temperature environment. At the same time, phase angle can also be used to evaluate the ratio of the viscous component and the elastic component. The lower phase angle shows better deformation recovery ability of asphalts.

**Figure 7:**
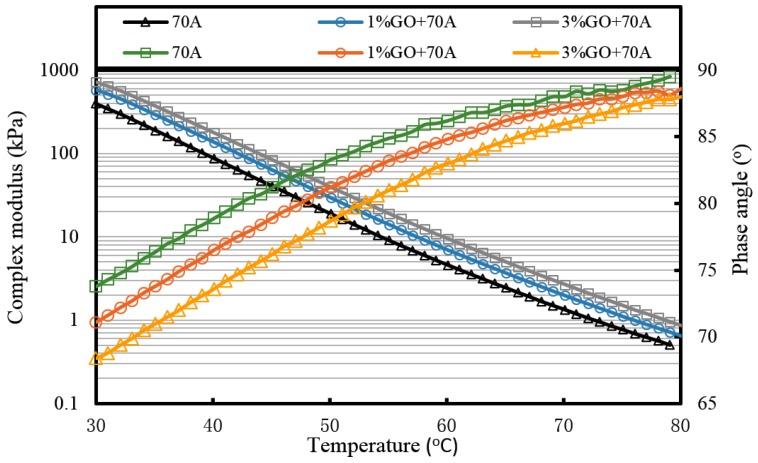
Complex modulus and phase angle of GO-modified 70A and its matrix at high temperature before aging.

**Figure 8:**
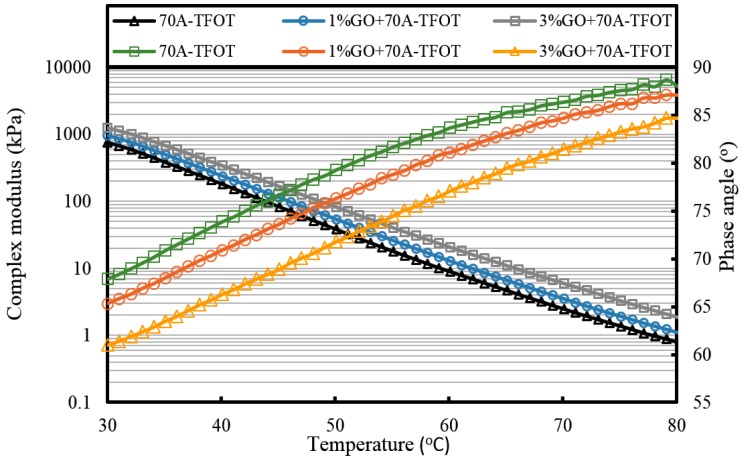
Complex modulus and phase angle of GO-modified 70A and its matrix at high temperature after TFOT aging.

**Figure 9:**
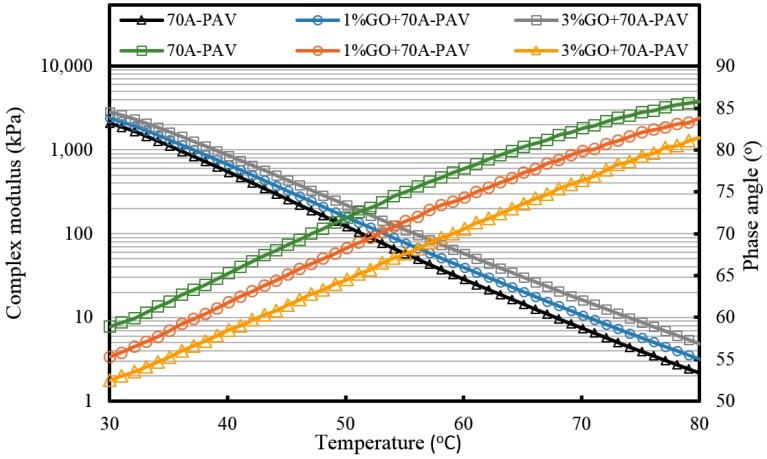
Complex modulus and phase angle of GO-modified 70A and its matrix at high temperature after PAV aging.

Figure 7, Figure 8 and Figure 9 and Table 2 present the complex modulus and phase angle of GO-modified 70A before and after aging.

It can be seen from Figure 7 that the complex modulus gradually increases and the phase angle reduces with the increasing weight ratio of GO. This means the introduction of GO can improve the high temperature properties of asphalt. After TFOT and PAV aging (seen from Figure 8 and Figure 9, and Table 2), the complex modulus and the phase angle exhibited the same regularity as samples before aging. The complex modulus of 3% GO-modified 70A was the largest, 1% GO-modified 70A was at the median, and 70A was the least. On the contrary, the phase angle of 3% GO-modified 70A was the least, 1% GO-modified 70A was at the median, and 70A was the largest. From Table 2, it can be seen that the complex modulus of 3% GO-modified 70A and 90A were about 1.8 times larger than 70A and 90A at 50 °C, respectively, after PAV aging. Moreover, the complex modulus of 3% GO-modified asphalts were 1.2 times larger than the base asphalts at 80 °C. The distinguished modulus of GO sheet could surely increase the complex modulus and reduce the phase angle. However, the anti-aging properties seemed unimproved by the introduction of GO sheet. The aging of asphalt leads to the increasing modulus and reducing phase angle. If GO slows down the oxidation of asphalt, the modulus of GO-modified 70A is lower than that of neat 70A after TFOT and PAV aging, or the modulus increment of GO-modified 70A is obviously smaller than neat 70A after aging. However, the increasing tendency of the modulus and reducing tendency of the phase angle for GO-modified 70A was almost the same as 70A after TFOT and PAV aging. Thus, from these statistics, GO could only improve the high-temperature properties, but not the anti-aging ability.

**Figure 10:**
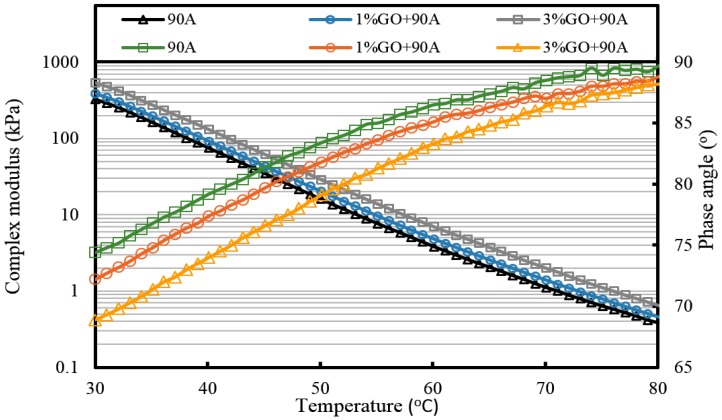
Complex modulus and phase angle of GO-modified 90A and its matrix at high temperature before aging.

**Figure 11:**
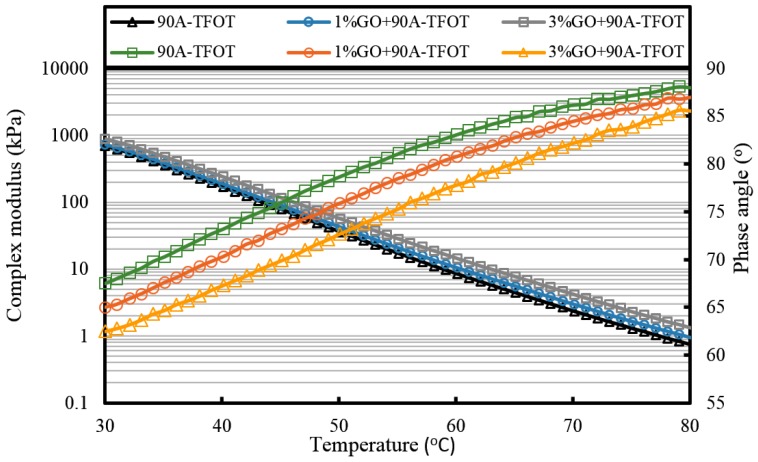
Complex modulus and phase angle of GO-modified 90A and its matrix at high temperature after TFOT aging.

**Figure 12:**
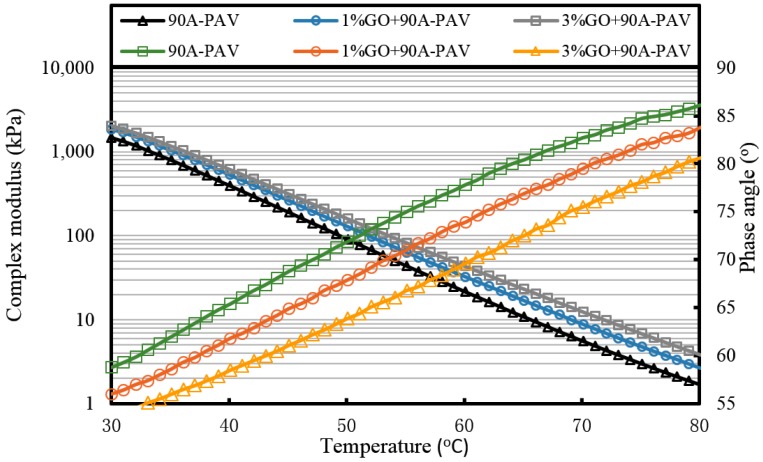
Complex modulus and phase angle of GO-modified 90A and its matrix at high temperature after PAV aging.

For GO-modified 90A (seen from Figure 10, Figure 11 and Figure 12, and Table 2), the results of the high-temperature sweep were the same as that of GO-modified 70A. The increasing modulus and reducing phase angle of all samples with the increasing addition of GO were also presented after aging. Thus, for both 70A and 90A, GO showed the same physical enhancement at high temperature. However, anti-aging improvement was not shown.

#### 3.3.2. Low-Temperature Sweep

At low temperature, asphalt is relatively like elastic materials. It becomes hard and brittle, which is the main reason that causes the cracking of asphalt pavement in winter. Therefore, the smaller complex modulus and larger phase angle mean better viscosity at low temperature.

**Figure 13:**
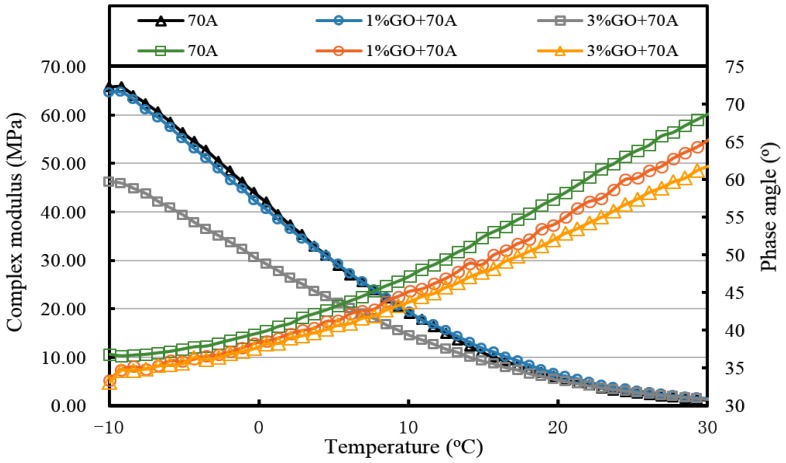
Complex modulus and phase angle of GO-modified 70A and its matrix at low temperature before aging.

**Figure 14:**
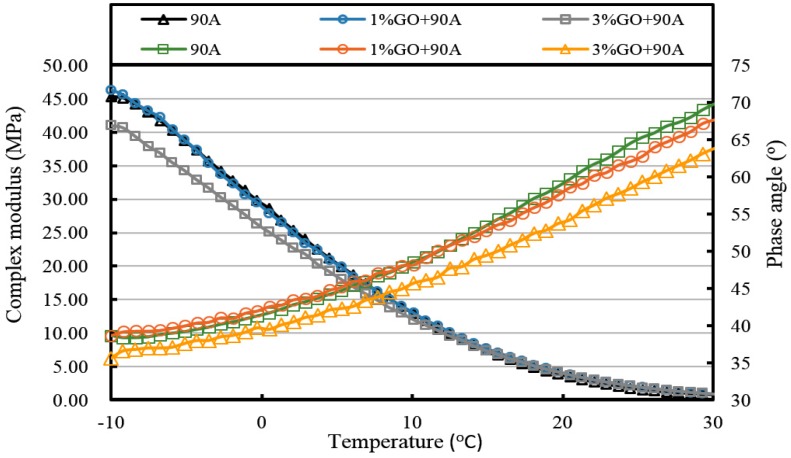
Complex modulus and phase angle of GO-modified 90A and its matrix at low temperature before aging.

**Figure 15:**
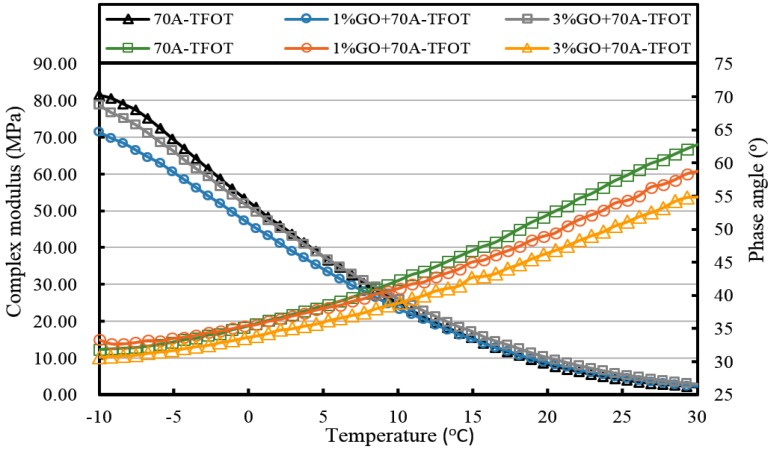
Complex modulus and phase angle of GO-modified 70A and its matrix at low temperature after TFOT aging.

**Figure 16:**
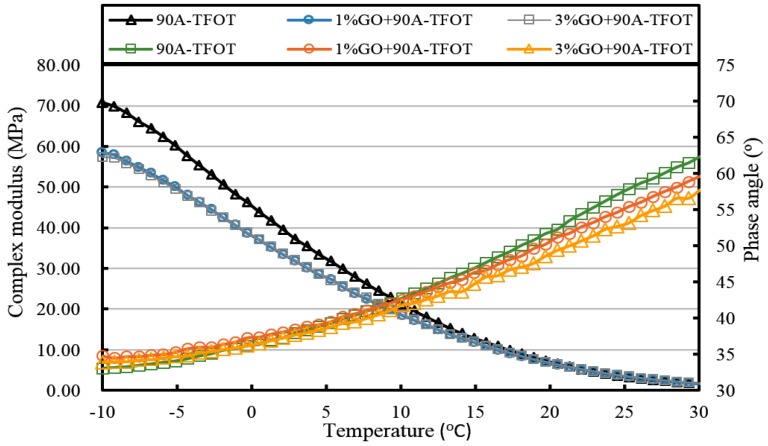
Complex modulus and phase angle of GO-modified 90A and its matrix at low temperature after TFOT aging.

**Figure 17:**
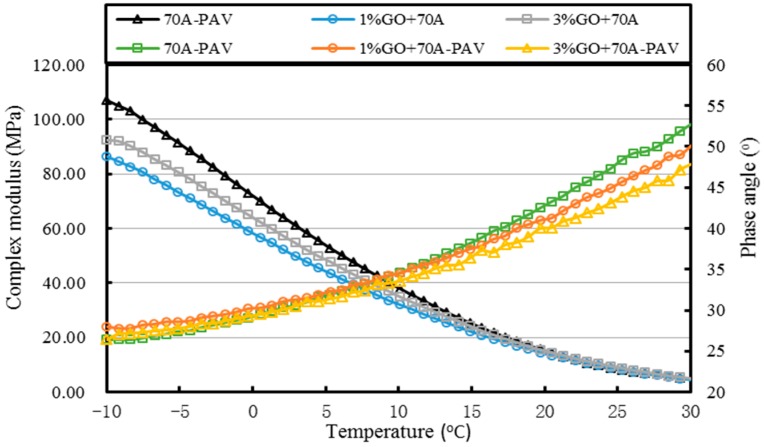
Complex modulus and phase angle of GO-modified 70A and its matrix at low temperature after PAV aging.

**Figure 18:**
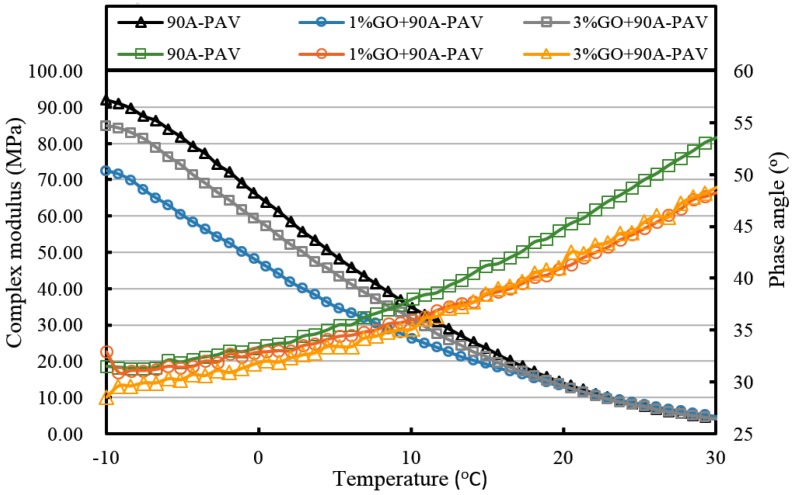
Complex modulus and phase angle of GO-modified 90A and its matrix at low temperature after PAV aging.

Figure 13, Figure 14, Figure 15, Figure 16, Figure 17 and Figure 18 and Table 3 show the complex modulus and phase angle of both modified and base asphalt at low temperature.

From Figure 13 and Figure 14 and Table 3, the introduction of 1% GO had a minor effect on the base asphalt. The complex modulus of 1% GO-modified 70A and 90A were almost overlapped compared to both base asphalts. When the content of GO rose to 3%, the complex modulus of modified 70A and 90A greatly reduced to 46.2 MPa and 41.0 MPa, respectively. For the phase angle, it was decreased by the addition of GO. However, the phase angle of 1% GO-modified 90A was slightly higher than the base asphalt below 10 °C (seen from Figure 14).

After TFOT aging (seen from Figure 15 and Figure 16, and Table 3), the complex modulus of 3% GO-modified 70A was slightly smaller than 70A below 5 °C and its phase angle was larger below 0 °C. Meanwhile, the complex modulus of 1% GO-modified 70A were significantly smaller and the phase angle was higher than that of base asphalt when temperature below 0 °C. From Figure 16, the complex modulus and phase angle of 1% and 3% GO-modified 90A were almost the same. Compared to 90A, the complex modulus of both modified asphalts was significantly smaller and the phase angle of them was slightly higher when the temperature was below 0 °C.

From Figure 17 and Figure 18, it can be seen clearly that the complex modulus of the both 1% GO-modified asphalts was lower than that of the both 3% GO-modified asphalts after PAV aging. Moreover, both base asphalts held the largest modulus. The phase angle of 70A was lower than 1% modified 70A under 10 °C, and lower than 3% modified asphalt under 0 °C. Meanwhile, the phase angle of 90A was larger than 1% GO-modified 90A, but the difference between them was not so obvious under 0 °C.

At the same time, it can be seen from statistics of Table 3 that the complex modulus of both 1% and 3% GO-modified 70A and 90A were almost the same as the two base asphalts at 20 °C. However, the complex modulus of GO-modified asphalts was much lower than the base asphalts at −10 °C. This means that the temperature sensitivity of GO-modified asphalts was better.

All of the complex modulus and phase angles of the modified asphalts and base asphalts showed the same fact that GO could improve the low temperature properties of asphalt before and after aging.

### 3.4. Py-GC/MS Test

**Figure 19:**
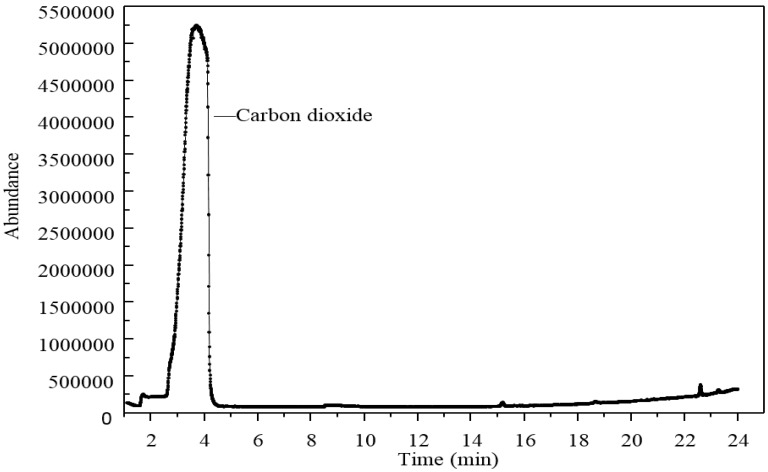
Gas chromatography of 3% GO-modified asphalt heated to 115 °C.

**Figure 20:**
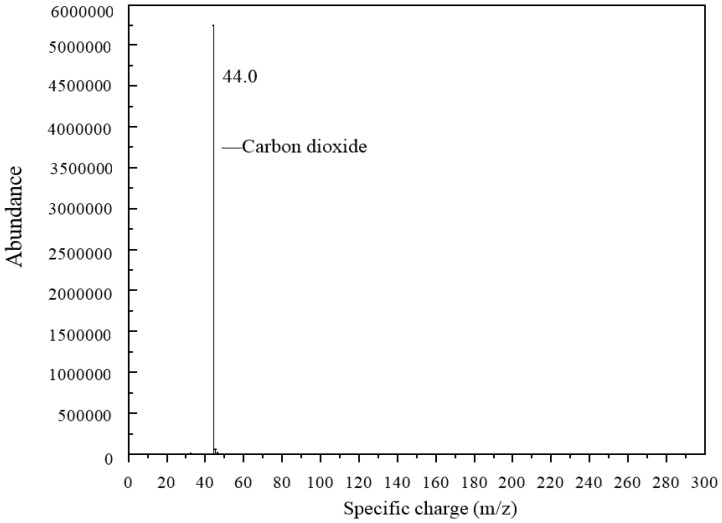
Mass spectrometry of 3% GO-modified asphalt heated to 115 °C.

Due to the boiling reaction that happened during the heating process, Py-GC/MS was applied to find out what types of gases were released from the 3% GO-modified asphalt. The results of the Py-GC/MS are shown in Figure 19 and Figure 20.

Figure 19 shows the gas chromatography results. Only one clear peak was displayed in this graph. This phenomenon meant only one type of gas was released when GO-modified asphalt was heated to 115 °C.

Figure 20 shows the mass spectrometry results. The gas released at 3.73 min at Py-GC/MS was applied to a mass spectrometry test. The specific charge of the tested gas was 44, and according to a mass spectrometry database, the gas was considered as carbon dioxide (CO_2_). This means that CO_2_ was released when GO-modified asphalt was heated to 115 °C.

### 3.5. Potential Application of GO-Modified Asphalt

The released CO_2_ gives the GO-modified asphalt some potential applications, like warm mix asphalt and flame retardant asphalt.

For example, water-containing technology and water-base technology are commonly applied to complete the foaming process in order to reduce the viscosity of asphalt [26,27,28]. The working principle of warm mix GO asphalt is just like the water-base technology. When asphalt is heated above 115 °C, a large amount of CO_2_ will be released. This can increase the volume of asphalt and decrease the mix viscosity of asphalt. Thus, the mixing temperature can be reduced.

In addition, GO holds the potential application as a flame retardant material on surface pavement. Thus, the reheating test of GO-modified asphalt was completed to investigate whether the release of CO_2_ would happen in this test. Firstly, the GO-modified asphalt was heated at 115 °C at which the boiling reaction occurred. Then, it was cooled to room temperature and the volume of the GO-modified asphalt returned to its original level. Thirdly, the sample was heated to 115 °C again and it was found that the CO_2_ gas was released again. Finally, the heat-cool-heat process was conducted for three cycles and the CO_2_ could still be released at the third cycle. Thus, supposing self-ignition of a car occurred, the fire would raise the surface pavement to a relatively high temperature. Once the temperature has reached above 115 °C, the GO-modified asphalt would release CO_2_ from the bottom of the enflamed car. Like a fire extinguisher set off from the bottom, it could retard the flame.

From the working principle, these two potential applications could work. However, more research should be completed to investigation and confirm this hypothesis.

## 4. Conclusions

GO was applied to modify two certain asphalts. The following items could be drawn from the above results:

1. GO could improve the high temperature property of base asphalt.
2. GO could also promote the low temperature property of base asphalt.
3. GO might increase the anti-aging property of base asphalt. However, the improving effects were not so obvious.
4. The addition of 1% GO performed better than the addition of 3% GO. Both 1% and 3% GO-modified asphalt could improve the high temperature properties, but for the low temperature property, 1% GO-modified asphalt was even better than 3% GO-modified asphalt. In addition, 1% introduction of GO costs three times less than 3% GO, with respect to economic considerations.

These conclusions are only limited to the materials used in this study and they may be different for other materials.

## Figures and Tables

**Table 1:** Physical properties of 70A and 90A.

Physical Properties	70A	90A
Softening point (°C)	49.8	47.8
Penetration (25 °C, 0.1 mm)	67.0	84.7
Ductility (10 °C, 1 cm)	>150	>150
Viscosity (60 °C, Pa·s)	210	204
Viscosity (135 °C, Pa·s)	0.56	0.47

**Table 2:** Complex modulus and phase angle of GO-modified asphalts and base asphalt before and after different aging processes.

Samples	Complex Modulus at 50 °C (kPa)	Phase Angle at 50 °C (°)	Complex Modulus at 80 °C (kPa)	Phase Angle at 80 °C (°)
70A	21.9	82.8	0.504	89.5
1% GO+70A	29.3	81.2	0.649	88.6
3% GO+70A	39.3	78.7	0.856	88
70A-TFOT	38	79.3	0.787	88
1% GO+70A-TFOT	53.9	76.3	1.1	87.1
3% GO+70A-TFOT	82.2	71.8	1.87	84.7
70A-PAV	121	71.9	2.16	85.8
1% GO+70A-PAV	158	68.3	3.17	83.9
3% GO+70A-PAV	218	64.5	4.79	81.5
90A	15.9	83.4	0.383	89.7
1% GO+90A	20.1	81.8	0.452	88.8
3% GO+90A	28.7	79.1	0.649	88.2
90A-TFOT	36.2	78.7	0.747	88
1% GO+90A-TFOT	42.7	75.9	0.95	87
3% GO+90A-TFOT	56.9	72.7	1.33	85.5
90A-PAV	90.5	71.9	1.68	86.1
1% GO+90A-PAV	129	67.9	2.65	83.8
3% GO+90A-PAV	160	63.9	3.85	80.6

**Table 3:** Complex modulus and phase angle of GO-modified asphalts and base asphalt before and after different aging processes.

Samples	Complex Modulus at −10 °C (MPa)	Phase Angle at −10 °C (°)	Complex Modulus at 20 °C (kPa)	Phase Angle at 20 °C (°)
70A	65.8	36.8	5.4	58.3
1% GO+70A	64.7	33.4	6.0	55
3% GO+70A	46.2	33.1	5.0	52.9
70A-TFOT	81.4	31.8	7.7	52.7
1% GO+70A-TFOT	71.4	33.3	8.1	49.4
3% GO+70A-TFOT	78.7	30.7	9.6	46.8
70A-PAV	107.0	26.4	14.4	43.3
1% GO+70A-PAV	86.4	28	13.4	41.3
3% GO+70A-PAV	92.4	26.4	14.4	40.1
90A	45.3	38.7	3.4	59.7
1% GO+90A	46.3	38.7	3.8	58.6
3% GO+90A	41.0	35.6	3.7	54.2
90A-TFOT	70.8	33.1	6.6	52.3
1% GO+90A-TFOT	58.5	34.8	6.3	51.2
3% GO+90A-TFOT	57.4	33.9	6.4	49.4
90A-PAV	92.0	31.5	13.3	45.3
1% GO+90A-PAV	72.4	32.9	12.4	41.3
3% GO+90A-PAV	84.8	28.5	12.5	42.5
